# Association of Glucose Fluctuations with Sarcopenia in Older Adults with Type 2 Diabetes Mellitus

**DOI:** 10.3390/jcm8030319

**Published:** 2019-03-06

**Authors:** Noriko Ogama, Takashi Sakurai, Shuji Kawashima, Takahisa Tanikawa, Haruhiko Tokuda, Shosuke Satake, Hisayuki Miura, Atsuya Shimizu, Manabu Kokubo, Shumpei Niida, Kenji Toba, Hiroyuki Umegaki, Masafumi Kuzuya

**Affiliations:** 1 Center for Comprehensive Care and Research on Memory Disorders, National Center for Geriatrics and Gerontology, Obu 474-8511, Japan; n-ogama@ncgg.go.jp (N.O.); toba@ncgg.go.jp (K.T.); 2 Department of Geriatric Medicine, National Center for Geriatrics and Gerontology, Obu 474-8511, Japan; kawashu@ncgg.go.jp (S.K.); satakes@ncgg.go.jp (S.S.); 3 Department of Community Healthcare and Geriatrics, Nagoya University Graduate School of Medicine, Nagoya 466-8550, Japan; umegaki@med.nagoya-u.ac.jp (H.U.); kuzuya@med.nagoya-u.ac.jp (M.K.); 4 Department of Cognition and Behavior Science, Nagoya University Graduate School of Medicine, Nagoya 466-8550, Japan; 5 Department of Endocrinology and Metabolism, National Center for Geriatrics and Gerontology, Obu 474-8511, Japan; hoisan@ncgg.go.jp (T.T.); tokuda@ncgg.go.jp (H.T.); 6 Department of Clinical Laboratory, National Center for Geriatrics and Gerontology, Obu 474-8511, Japan; 7 Medical Genome Center, National Center for Geriatrics and Gerontology, Obu 474-8511, Japan; sniida@ncgg.go.jp; 8 Department of Home Care Coordinators, National Center for Geriatrics and Gerontology, Obu 474-8511, Japan; hmiura@ncgg.go.jp; 9 Department of Cardiology, National Center for Geriatrics and Gerontology, Obu 474-8511, Japan; ashimizu@ncgg.go.jp (A.S.); mkokubo@ncgg.go.jp (M.K.); 10 Institutes of Innovation for Future Society, Nagoya University, Nagoya 464-8601, Japan

**Keywords:** Alzheimer’s disease, type 2 diabetes mellitus, glucose fluctuations, sarcopenia

## Abstract

Type 2 diabetes mellitus accelerates loss of muscle mass and strength. Patients with Alzheimer’s disease (AD) also show these conditions, even in the early stages of AD. The mechanism linking glucose management with these muscle changes has not been elucidated but has implications for clarifying these associations and developing preventive strategies to maintain functional capacity. This study included 69 type 2 diabetes patients with a diagnosis of cognitive impairment (*n* = 32) and patients with normal cognition (*n* = 37). We investigated the prevalence of sarcopenia in diabetes patients with and without cognitive impairment and examined the association of glucose alterations with sarcopenia. Daily glucose levels were evaluated using self-monitoring of blood glucose, and we focused on the effects of glucose fluctuations, postprandial hyperglycemia, and the frequency of hypoglycemia on sarcopenia. Diabetes patients with cognitive impairment displayed a high prevalence of sarcopenia, and glucose fluctuations were independently associated with sarcopenia, even after adjusting for glycated hemoglobin A1c (HbA1c) levels and associated factors. In particular, glucose fluctuations were significantly associated with a low muscle mass, low grip strength, and slow walking speed. Our observation suggests the importance of glucose management by considering glucose fluctuations to prevent the development of disability.

## 1. Introduction

The definition and diagnosis of sarcopenia were updated by the European Working Group on Sarcopenia in Older People [1]. The revised consensus focuses on the importance of promoting early detection and treatment of sarcopenia [1]. Because sarcopenia predicts adverse outcomes such as chronic disease progression, mortality, and functional disability [2], developing strategies to prevent sarcopenia in older adults is necessary.

Older adults with type 2 diabetes have a higher prevalence of sarcopenia than non-diabetic individuals [3,4]. Some studies have reported that increased glycated hemoglobin A1c (HbA1c) levels are associated with impaired muscle quality, muscle strength and physical performance [5,6,7]. However, HbA1c levels do not adequately reflect the mean glucose concentration and are not associated with hypoglycemic risk in diabetes patients [8,9]. Severe hypoglycemic events lead to deterioration in general health, resulting in an increased risk of mobility disabilities [10]. Therefore, continuous assessments of daily glucose excursions are needed to determine the association of glucose management with sarcopenia.

Muscle changes and physical dysfunctions are observed in the early stages of Alzheimer’s disease (AD) [11,12]. Lean mass is reduced in early AD and is associated with brain atrophy [11]. Furthermore, the nutritional status is correlated with regional cerebral glucose metabolism in prodromal AD, and the prevalence of sarcopenia increases with the degree of cognitive decline [13,14]. These studies suggest that AD-related degenerative pathologies have a negative impact on muscle structure and physical function. Additionally, AD patients with diabetes have a variety of difficulties in glucose management and therefore might have a higher prevalence of sarcopenia than diabetes patients without AD. However, to date, the prevalence of sarcopenia in diabetes patients with and without cognitive impairment remains unclear. Furthermore, the association of glucose management with sarcopenia in these patients is also unknown.

The aims of this study were as follows: (1) to clarify the prevalence of sarcopenia in older type 2 diabetes patients with cognitive impairment or normal cognition, and (2) to clarify the association of glucose management with sarcopenia in these patients. We hypothesized that diabetes patients with cognitive impairment would display a high prevalence of sarcopenia and that abnormal glucose profiles would be associated with muscle changes and physical dysfunction.

## 2. Methods

### 2.1. Participants

The study was conducted in accordance with the Declaration of Helsinki, and the protocol was approved by the Ethics Committee of the National Center for Geriatrics and Gerontology (NCGG) (approval no. 682). All participants provided written informed consent before participating in the study. We included 69 outpatients (cognitive impairment: *n* = 32; normal cognition: *n* = 37) who visited the NCGG hospital from 2014 to 2016. The presence of cognitive impairment was defined as probable or possible AD and amnestic mild cognitive impairment (aMCI) according to the criteria of the National Institute on Aging–Alzheimer’s Association workgroups and the definition provided by Petersen et al. [15,16]. Persons with NC attended NCGG hospital for suspected memory impairment but were assessed as having normal cognition. Patients meeting all the following criteria were included in this study: (1) outpatients with type 2 diabetes treated with antidiabetic agents; (2) aged 65 years or older; (3) living in their houses; (4) with families or caregivers who support self-monitoring of blood glucose (SMBG); and (5) a Mini-Mental State Examination (MMSE) score ≥10 for cognitive impairment. The exclusion criteria were as follows: (1) severe hearing loss and visual impairment; (2) severe health conditions, such as cardiac failure, renal disorder or liver dysfunction; and (3) neurological disorders other than AD or aMCI.

### 2.2. Assessment of Clinical Parameters and Comorbidities Associated with Diabetes

Clinical data and blood samples were distributed from the Biobank, which collects and stores biological material and associated clinical data for biomedical research. Data on the diagnosis, antidiabetic medication use, and polypharmacy (defined as taking five or more types of oral medicine) [17] were obtained from clinical charts. Global cognitive function was assessed by the MMSE [18]. Diabetes-associated complications were evaluated for the coexistence of neuropathy, retinopathy and nephropathy [19]. Diabetic neuropathy was defined as either the loss of Achilles tendon reflex or the presence of neuropathic symptoms. Diabetic retinopathy was fundoscopically assessed through dilated pupils by experienced ophthalmologists. Diabetic nephropathy was defined as an albumin-to-creatinine ratio >300 µg/mg or a urinary protein concentration >0.5 mg/dL. We obtained information on the following biochemical parameters: HbA1c, triglyceride, total cholesterol, high-density lipoprotein cholesterol, low-density lipoprotein cholesterol, estimated glomerular filtration rate, serum albumin, and urinary albumin. HbA1c levels were expressed in the National Glycohemoglobin Standardization Program units.

### 2.3. Measurement of Daily Glucose Levels

Daily glucose levels were recorded by SMBG (MS-FR201B; Terumo Corp., Tokyo, Japan) and were measured at five time points per day (05:00 h, before breakfast, 2 h after breakfast, before lunch, and before dinner) on eight separate days during a two-month period. Glucose levels usually nadir early in the morning and before each meal, and postprandial glucose levels usually peak at 2 h after breakfast [20]. Therefore, we evaluated the glucose levels at these time points. When diabetic patients with cognitive impairment used SMBG, their families helped with the measurement. Hypoglycemia was defined as a glucose level ≤70 mg/dL [21], and the presence or absence of hypoglycemic symptoms was recorded at every SMBG measurement point. Glucose fluctuations were determined based on the diurnal range from minimum glucose levels to maximum glucose levels.

### 2.4. Evaluation of Sarcopenia

Sarcopenia was defined as low muscle mass plus low muscle strength and/or low physical performance according to the Asian Working Group for Sarcopenia (AWGS) [22]. Low muscle mass was defined as a calf circumference <31 cm [23], which was measured with the patient in the supine position with the left knee raised at a right angle from the thigh. Calf circumference was correlated with appendicular skeletal muscle mass measured by dual-energy X-ray absorptiometry and has been proposed as a surrogate marker of muscle mass for diagnosing sarcopenia [24]. Low muscle strength was determined by low hand grip strength (<26 kg in men and <18 kg in women). Hand grip strength was measured with a Smedley dynamometer (Matsumiya Medical Instruments, Tokyo, Japan). Low physical performance was assessed by slow walking speed. The presence of slow walking speed was defined as individuals who answered “No” to the following question: "Can you cross the road within the green signal interval?" [25].

### 2.5. Statistical Analysis

All analyses were performed using SPSS for Windows version 22.0 (IBM Corp., Armonk, NY, USA). The distributions of data were assessed for normality using the Shapiro–Wilk test. Differences in demographics between diabetic patients with cognitive impairment and patients with normal cognition were examined by unpaired *t*-tests (for parametric variables) or the Mann-Whitney U test (for non-parametric variables). Categorical variables were analyzed by the chi-squared test or Fisher’s exact test. Differences in glucose levels with and without sarcopenia were analyzed using unpaired *t*-tests or the Mann–Whitney U test. To identify the factors associated with sarcopenia, we performed logistic regression analysis. First, we conducted stepwise analysis to select the most influential glucose index for sarcopenia. Next, we conducted logistic regression with forward variable selection to construct a model based on the variables associated with sarcopenia, defined as those with *p* < 0.05, adjusted for factors related to the development of sarcopenia (i.e., age, HbA1c level and the presence of diabetic neuropathy) [4,5,7]. In this analysis, we calculated the adjusted odds ratio (OR) and 95% confidence interval (CI) for factors associated with sarcopenia. The dependent variable was sarcopenia, and the independent variable was the glucose index, which was selected as the most influential variable associated with sarcopenia in a first regression analysis. Statistical significance was set at *p* < 0.05.

## 3. Results

### 3.1. Clinical Characteristics of the Study Participants

The demographics of the participants are shown in Table 1. No differences in age, sex, body mass index, diabetic comorbidities or medication use were found between the diabetic patients with cognitive impairment and the normal cognition group. The cognitive impairment group had a lower MMSE score than the normal cognition group. Serum triglyceride levels were higher and albumin concentrations were lower in the cognitive impairment group, but the other metabolic markers did not differ between the groups. Regarding the average glucose levels during the 2 months of study, the glucose level before lunch was high in the cognitive impairment group. The prevalence of sarcopenia, represented by a low muscle mass, low grip strength and slow walking speed, was higher in the cognitive impairment group than that in the normal cognition group.

### 3.2. Differences in Glucose Profiles According to Sarcopenia

**Figure 1:**
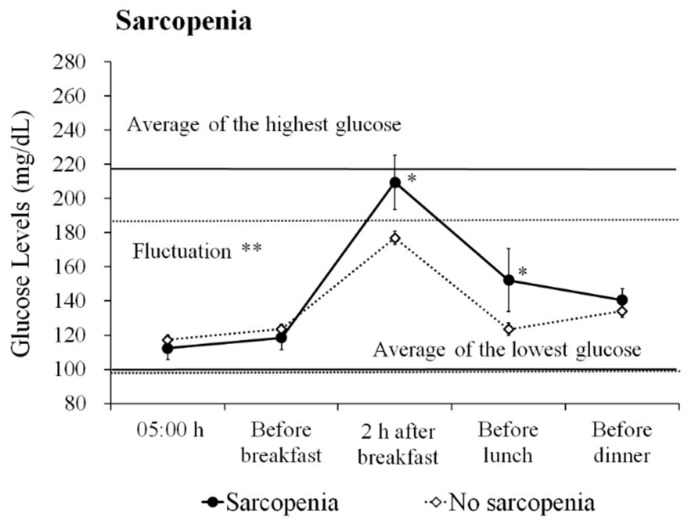
Daily glucose profiles based on sarcopenia. The figure shows the average ± standard error (SE) glucose level based on sarcopenia in all participants. Average of the highest glucose level: the average of the maximum glucose level of the day during the measurement period. Average of the lowest glucose level: the average of the minimum glucose level of the day during the measurement period. The solid line represents the sarcopenia group, and the dotted line indicates the no sarcopenia group. Fluctuation: the average of the diurnal range from the minimum glucose level to the maximum glucose level. ** *p* < 0.01, * *p* < 0.05.

First, we examined the differences in glucose profiles with and without sarcopenia in all subjects (Figure 1). Patients with sarcopenia displayed larger fluctuations in daily glucose levels compared to non-sarcopenia patients (117.3 mg/dL vs. 88.0 mg/dL, *p* = 0.005). In addition, patients with sarcopenia showed high glucose levels 2 h after breakfast and before lunch (209.5 mg/dL vs. 177.0 mg/dL, *p* = 0.011, and 152.2 mg/dL vs. 123.4 mg/dL, *p* = 0.037, respectively).

Next, we examined the glucose profile differences with and without sarcopenia in the cognitive impairment group (Figure A1). Cognitive impairment patients with sarcopenia displayed large glucose fluctuations (119.5 mg/dL vs. 90.7 mg/dL, *p* = 0.021) and elevated glucose levels 2 h after breakfast (210.6 mg/dL vs. 177.4 mg/dL, *p* = 0.044). In the normal cognition group, because only one patient was diagnosed with sarcopenia, we could not examine the association between glucose profiles and sarcopenia.

### 3.3. Differences in Glucose Profiles Based on Sarcopenia Components

**Figure 2:**
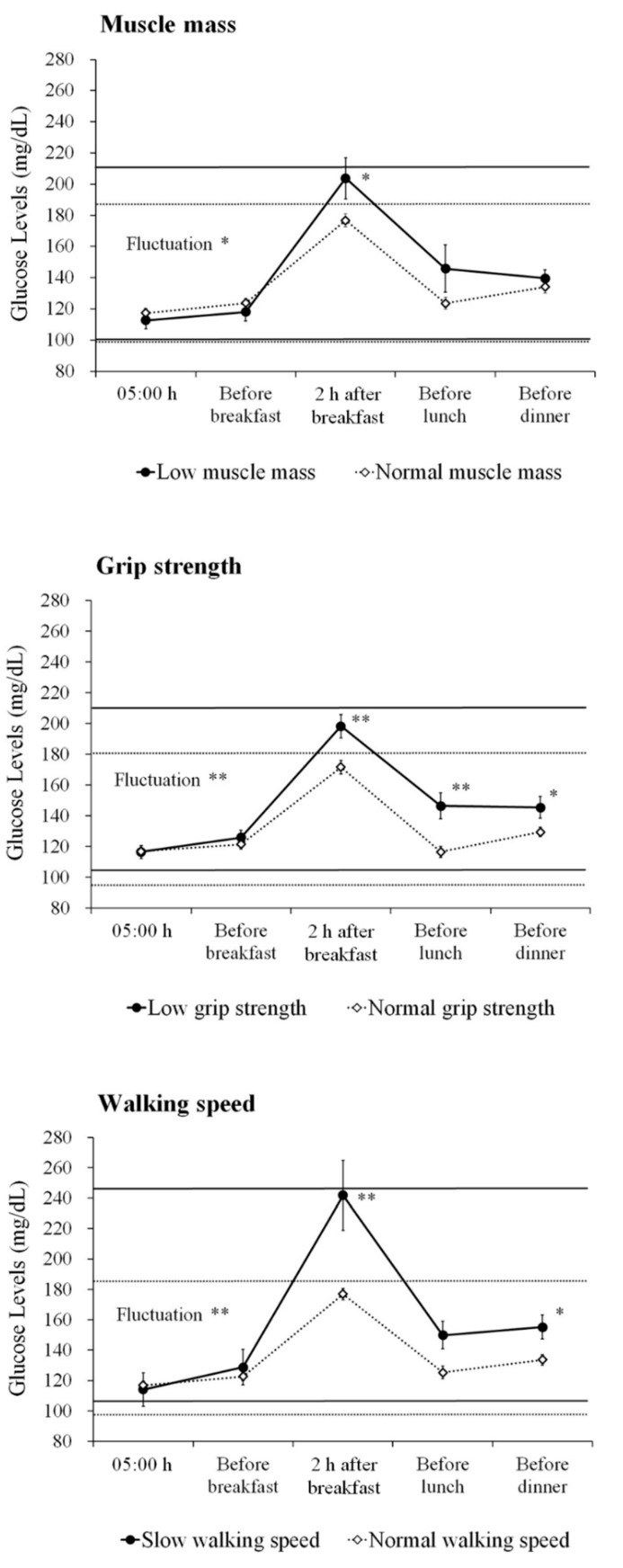
Daily glucose profiles based on the sarcopenia components. The figures show the average ± SE glucose level based on the components of sarcopenia in all participants. ** *p* < 0.01, * *p* < 0.05.

**Figure A1:**
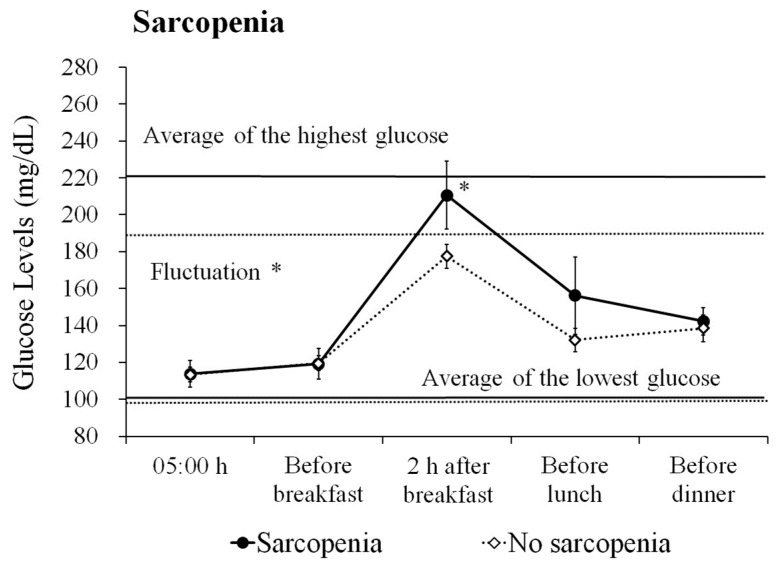
Daily glucose profiles based on sarcopenia in the cognitive impairment group. The figure shows the average ± standard error (SE) glucose level. Average of the highest glucose level: the average of the maximum glucose level of the day during the measurement period. Average of the lowest glucose level: the average of the minimum glucose level of the day during the measurement period. The solid line represents the sarcopenia group, and the dotted line indicates the no sarcopenia group. Fluctuation: the average of the diurnal range from the minimum glucose level to the maximum glucose level. * *p* < 0.05.

The differences in glucose profiles with and without sarcopenia components in all subjects are shown in Figure 2. Patients with a low muscle mass, low grip strength and slow walking speed displayed larger glucose fluctuations compared to patients without these conditions (low muscle mass: 110.8 mg/dL vs. 88.1 mg/dL, *p* = 0.018; low grip strength: 104.4 mg/dL vs. 84.4 mg/dL, *p* = 0.005; and slow walking speed: 136.6 mg/dL vs. 88.6 mg/dL, *p* = 0.003, respectively). Furthermore, patients with these conditions displayed high glucose levels 2 h after breakfast (low muscle mass: 203.8 mg/dL vs. 176.8 mg/dL, *p* = 0.020; low grip strength: 198.1 mg/dL vs. 171.5 mg/dL, *p* = 0.002; and slow walking speed: 241.8 mg/dL vs. 177.0 mg/dL, *p* < 0.001, respectively). In addition, patients with low grip strength showed high glucose levels before lunch (146.2 mg/dL vs. 116.4 mg/dL, *p* = 0.001) and before dinner (145.3 mg/dL vs. 129.4 mg/dL, *p* = 0.034), and patients with a slow walking speed showed high glucose levels before dinner (155.2 mg/dL vs. 133.7 mg/dL, *p* = 0.041).

**Figure A2:**
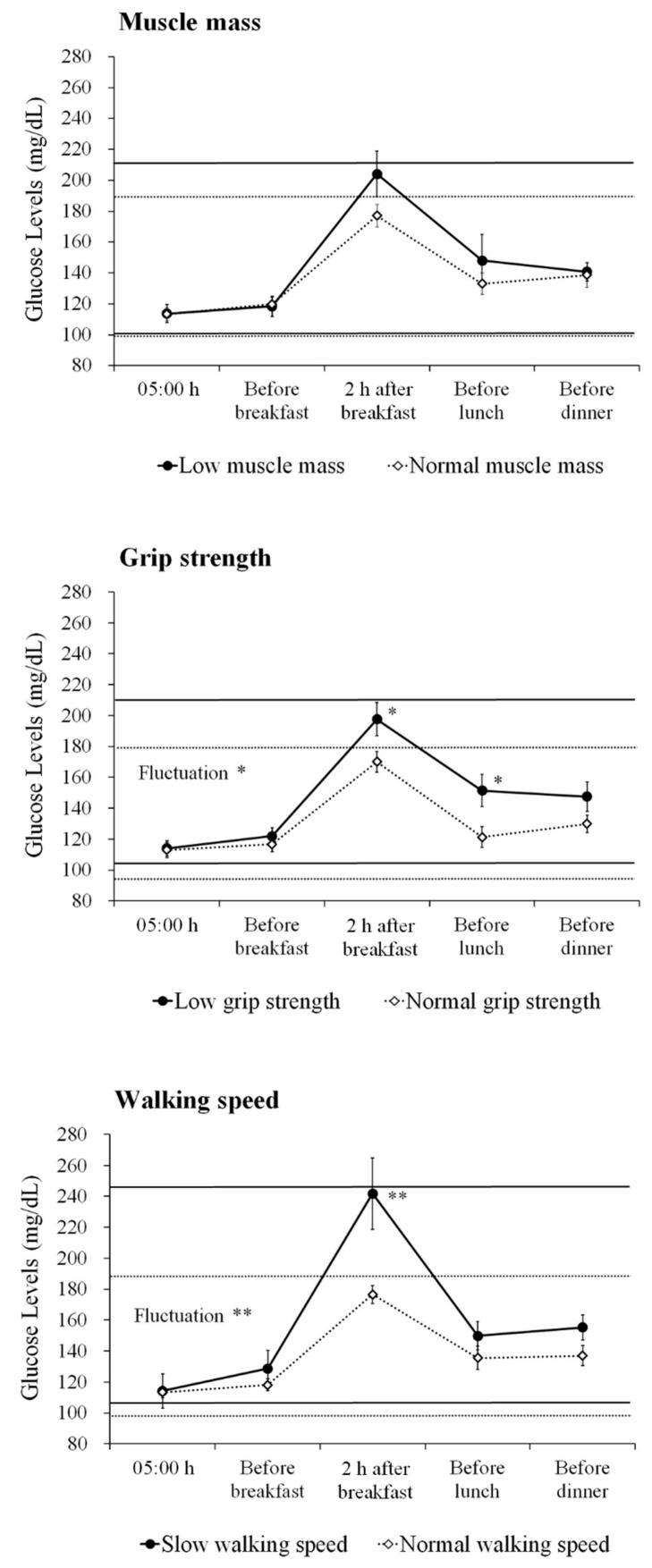
Daily glucose profiles based on the sarcopenia components in the cognitive impairment group. The figures show the average ± SE glucose level based on the components of sarcopenia. ** *p* < 0.01, * *p* < 0.05.

In the cognitive impairment group, patients with a low muscle mass tended to have large glucose fluctuations and high glucose levels 2 h after breakfast, but this difference did not reach statistical significance (111.9 mg/dL vs. 91.2 mg/dL, *p* = 0.076, and 204.1 mg/dL vs. 177.1 mg/dL, *p* = 0.077, respectively) (Figure A2). Patients with low grip strength displayed large glucose fluctuations and high glucose levels 2 h after breakfast and before lunch (107.4 mg/dL vs. 85.2 mg/dL, *p* = 0.033, 197.6 mg/dL vs. 170.0 mg/dL, *p* = 0.038, and 151.4 mg/dL vs. 121.3 mg/dL, *p* = 0.025, respectively). In addition, patients with a slow walking speed displayed large glucose fluctuations and high glucose levels 2 h after breakfast (136.6 mg/dL vs. 91.4 mg/dL, *p* = 0.009, and 241.8 mg/dL vs. 176.5 mg/dL, *p* = 0.001, respectively).

### 3.4. Prevalence of Hypoglycemia and its Association with Sarcopenia

Hypoglycemia (glucose range: 43–70 mg/dL) was observed at 49 measurement points (1.78% of all measurement points) in all subjects. Severe hypoglycemic symptoms or events were not observed in this study. Typical hypoglycemic symptoms, such as hand tremor and palpitations, were observed in one normal cognition subject whose glucose level was 45 mg/dL. Hypoglycemia-related symptoms, such as nausea, dizziness, light-headedness, blurred vision and fatigue, were reported at four measurement points in the cognitive impairment group and four measurement points in the normal cognition group.

There was no difference in the frequency of hypoglycemia between patients with and without sarcopenia in all subjects (1.3 ± 2.5 vs. 0.6 ± 1.1, *p* = 0.884, Mann–Whitney U test). The effect of mild hypoglycemia (glucose level ≤80 mg/dL or ≤90 mg/dL) on sarcopenia was also examined. However, there was no difference in the frequency of mild hypoglycemia among the patients (glucose level ≤80 mg/dL; 2.4 ± 3.9 vs. 1.8 ± 2.5, *p* = 0.922, glucose level ≤90 mg/dL; 4.9 ± 6.8 vs. 4.6 ± 4.7, *p* = 0.741, Mann–Whitney U test).

We conducted the same analysis in the cognitive impairment group. However, no difference was found in the frequency of hypoglycemia between patients with and without sarcopenia.

### 3.5. Association of Glucose Indices with Sarcopenia

Because the patients with sarcopenia displayed large glucose fluctuations, high glucose levels at 2 h after breakfast and before lunch, we conducted a regression analysis using the stepwise method to extract the most influential glucose index on sarcopenia. The results revealed glucose fluctuations as the most influential variable on sarcopenia (OR = 1.041, *p* = 0.012). Therefore, we used glucose fluctuations as an independent variable for the subsequent logistic regression analysis.

After adjusting for age, HbA1c level and the presence of diabetic neuropathy, glucose fluctuations were independently associated with sarcopenia (Table 2). Additionally, among the components of sarcopenia, glucose fluctuations were significantly associated with low muscle mass, low grip strength, and slow walking speed.

In the cognitive impairment group, glucose fluctuations were independently associated with sarcopenia (OR = 1.038, *p* = 0.035). Furthermore, glucose fluctuations were significantly associated with low grip strength (OR = 1.034, *p* = 0.049) and slow walking speed (OR = 1.077, *p* = 0.023). The association between glucose fluctuation and low muscle mass did not reach statistical significance, which may have been caused by the low statistical power due to the small sample size.

## 4. Discussion

The present study revealed that diabetes patients with cognitive impairment had a high prevalence of sarcopenia. Glucose fluctuations were independently associated with sarcopenia after adjusting for HbA1c levels and associated factors. Our observation suggests the importance of glucose management by considering glucose fluctuations in older adults with diabetes.

In the current study, the prevalence of sarcopenia was higher in the cognitive impairment group than the normal cognition group. However, diabetes comorbidities and medication were comparable between the groups. Regarding the underlying mechanisms of sarcopenia in diabetes, insulin resistance in peripheral tissues has been suggested [3,26]. Insulin resistance is associated with impaired mitochondrial function in muscles, leading to the production of oxidative damage [3]. In addition, increased levels of inflammatory cytokines, such as interleukin (IL)-1, IL-6, and tumor necrosis factor alpha (TNF-α), are observed in diabetes but are also found in AD patients [27]. These factors are closely related to decreased muscle function [28]. Furthermore, anabolic hormones play a major role in muscle integrity; testosterone decline is found in diabetes patients and also involved in the development of AD [3,29]. More recently, AD-related brain pathology has also been associated with low physical performance [30,31]. Thus, diabetes patients with cognitive impairment have multiple factors that may contribute to the development of sarcopenia.

In this investigation, we found an independent association between glucose fluctuations and sarcopenia in cognitively impaired patients. Acute hyperglycemia increases Aβ production, and altering insulin signaling lead to changes in Aβ levels in the brain [32,33,34]. Increased levels of Aβ have been also found in the skeletal muscle, which may cause impaired peripheral glucose metabolism [35,36]. In addition, glucose fluctuations are a greater trigger of oxidative stress than sustained hyperglycemia, and independently contribute to the development of microvascular complications [37,38]. Because brain tissue of AD show increased oxidative stress during the course of the disease [39], AD patients are more likely to be susceptible to the influence of glucose fluctuations on the brain. In fact, our previous study showed that glucose fluctuations during postprandial periods were independently associated with frontal white matter hyperintensity (WMH) in diabetic patients with AD but not in patients with normal cognition [40]. Frontal WMH is known to play a predominant role in motor dysfunctions in AD/aMCI patients [41,42]. Furthermore, an association between elevated 2 h post-load glucose levels evaluated by the oral glucose tolerance test and brain atrophy has also been reported [43]. Thus, brain structural changes associated with glucose fluctuations might further contribute to mobility disabilities in diabetes patients with cognitive impairment.

In this study, the prevalence of sarcopenia was lower than previously reported [3], particularly in the normal cognition group. This can be explained by differences in the measurement method for muscle mass in the current study. Low muscle mass is an essential condition for the AWGS definition of sarcopenia and was measured by calf circumference. A previous study showed that fat-free mass decreases and fat mass increases with aging [44]. However, in older adult with diabetes, muscle mass is even lower than in non-diabetic individuals [45]. Furthermore, higher level of insulin resistance has been associated with low muscle mass and high fat mass [46]. A study using peripheral quantitative computed tomography showed that diabetes patients have a larger intramuscular and intermuscular adipose tissue in calf muscles than individuals without diabetes [47]. Therefore, diabetic patients are considered to have deteriorated muscle quality compared to individuals without diabetes. It seems likely that these muscle changes cannot be correctly measured with calf circumference. Although, calf circumference is considered suitable for evaluating muscle mass, our observations suggest that calf circumference may be difficult to accurately estimate the muscle mass of patients with diabetes.

We found no association between hypoglycemia and sarcopenia. Diabetes patients who experienced hypoglycemic events had a high prevalence of comorbidities, disabilities, and malnutrition, and were often treated with insulin therapy and use of polypharmacy [10]. However, in this study, no difference was identified in these parameters between patients with and without hypoglycemia. The study participants had relatively well-controlled glucose levels, and severe hypoglycemic events were not observed. Therefore, we could not detect a sufficient impact of hypoglycemia. In fact, hospitalization due to severe hypoglycemia can cause further deterioration in physical function [10]. Hypoglycemic symptoms in older patients are mild or asymptomatic, and episodes are less likely to manifest [10]. We further addressed concerns about repeated mild hypoglycemia but found no association of mild hypoglycemia with sarcopenia.

Currently, sarcopenia and frailty are considered a third category of diabetes-related complications in addition to the traditional microvascular and macrovascular disease [4]. Because muscle weakness and impaired physical function resulting from sarcopenia are major physiological components of frailty, these conditions often overlap [3,48]. Some studies reported that frailty and cognitive decline are reciprocally related and suggested that they share underlying pathophysiological mechanisms, including AD pathology, chronic inflammation, mood disorder, and co-morbidities [49,50,51]. Thus, patients with cognitive impairment are at high risk of vulnerability due to diverse extrinsic and intrinsic conditions. Recently, some types of interventions, such as physical exercise, nutritional therapy, and multicomponent interventions, to prevent frailty have been attempted [52]. Exercise training has benefits for physical capacity and cognitive performance [53], improves skeletal muscle insulin sensitivity and increases muscle mass in frail older adults [54]. Furthermore, the benefits of resistance training in improving glycemic status and muscle strength have been reported [55]. Therefore, in addition to glucose management using medications appropriately to avoid glucose fluctuations, multimodal interventions would be beneficial to delay or prevent disability in older diabetic patients.

This study has several limitations. First, because this study had a cross-sectional design, causal relationships should be carefully considered. Second, the sample size was relatively small. Nevertheless, our results revealed the independent association between glucose fluctuations and sarcopenia, even after adjusting for several confounding factors. Furthermore, our sample size was largely comparable to that of a previous study that identified an association between altered glucose dynamics and frailty [56]. Additional studies with a large cohort estimated by calculation of the appropriate sample size are needed to confirm our findings. Third, the cognitive impairment group was assigned based on the criteria of probable or possible AD and aMCI [15,16], but biomarkers for AD pathology were not assessed in this study. A biological definition based on neuroimaging with positron emission tomography and cerebrospinal fluid biomarkers is necessary to achieve a precise diagnosis [57,58]. Fourth, SMBG was used evaluate glucose levels, but continuous glucose monitoring (CGM) might more accurately represent the glucose profiles [59]. However, CGM in patients with cognitive impairment is difficult. Because we measured glucose levels on 8 separate days during the two-month period, our data could reflect the characteristics of glucose profiles over this period. Finally, we did not use elaborate equipment to evaluate muscle mass and walking speed. It is suitable to measure muscle mass using bioimpedance analysis [22]. Regarding walking speed, our evaluation was based on self-reported answers related to road crossing. To safely cross the road while the traffic signal is green, a pedestrian walking speed of 1.2 m/s is required [60]. This value is the same as the cut-off for slow walking speed in Japanese elderly individuals [22].

## 5. Conclusions

The prevalence of sarcopenia was high in diabetes patients with cognitive impairment. Glucose fluctuations were independently associated with sarcopenia, particular with low muscle mass, low grip strength and slow walking speed. Our study described the characteristic glucose profiles in older diabetes patients with sarcopenia. To maintain functional ability in older diabetes patients, confirmation of our data in future longitudinal studies with a larger cohort is required.

## Figures and Tables

**Table 1:** Clinical characteristics of the study participants.

	Total (*n* = 69)		Cognitive Impairment (*n* = 32)		Normal Cognition (*n* = 37)		*p*-Value ^†^
	Mean (SD) or *n* (%)	Min–Max	Mean (SD) or *n* (%)	Min–Max	Mean (SD) or *n* (%)	Min–Max	
Age, years	75.0 (5.3)	65–87	76.0 (5.8)	65–87	74.2 (4.7)	65–83	0.146
Male, *n* (%)	36 (52.2)		15 (46.9)		21 (56.8)		0.413
Body mass index, kg/m^2^	23.8 (2.7)	17.8–31.0	23.6 (2.6)	17.8–29.4	24.0 (2.7)	19.9–31.0	0.597
Mini-Mental State Examination	24.4 (5.0)	13–30	21.0 (5.2)	13–29	27.4 (2.0)	22–30	<0.001
**Diabetes and comorbidities**							
Duration of diabetes, years	15.3 (10.8)	2–48	15.3 (10.6)	2–40	15.4 (11.0)	2–48	0.899
Diabetic neuropathy, *n* (%)	45 (65.2)		22 (68.8)		23 (62.2)		0.567
Diabetic retinopathy, *n* (%)	16 (23.2)		5 (15.6)		11 (29.7)		0.166
Diabetic nephropathy, *n* (%)	21 (30.4)		12 (37.5)		9 (24.3)		0.236
Coronary artery disease, *n* (%)	15 (21.7)		6 (18.8)		9 (24.3)		0.576
Hypertension, *n* (%)	53 (76.8)		24 (75.0)		29 (78.4)		0.740
**Medications and antidiabetic agents, *n* (%)**							
Polypharmacy	56 (81.2)		29 (90.6)		27 (73.0)		0.061
Biguanide	20 (29.0)		10 (31.3)		10 (27.0)		0.700
Thiazolidine	8 (11.6)		6 (18.8)		2 (5.4)		0.132
DPP4 inhibitor	49 (71.0)		23 (71.9)		26 (70.3)		0.884
Sulfonylurea	40 (58.0)		19 (59.4)		21 (56.8)		0.826
Insulin secretion promoter	2 (2.9)		2 (6.3)		0 (0.0)		0.211
α-Glucosidase inhibitor	16 (23.2)		7 (21.9)		9 (24.3)		0.810
Insulin	13 (18.8)		7 (21.9)		6 (16.2)		0.549
GLP-1 receptor agonists	2 (2.9)		1 (3.1)		1 (2.7)		1.000
***Biochemical parameters***							
HbA1c, %	7.1 (0.6)	6.2–9.3	7.3 (0.7)	6.2–9.3	7.0 (0.5)	6.3–8.6	0.107
Triglyceride, mg/dL	139.8 (69.4)	44–330	165.5 (72.1)	65–330	117.6 (57.2)	44–279	0.004
Total cholesterol, mg/dL	190.3 (41.1)	108–316	192.0 (41.1)	108–316	188.9 (41.6)	137–309	0.524
HDL cholesterol, mg/dL	53.6 (13.7)	27–92	50.8 (13.6)	27–83	56.0 (13.5)	37–92	0.112
LDL cholesterol, mg/dL	109.2 (36.0)	46–238	109.5 (37.2)	46–211	108.9 (35.4)	67–238	0.928
eGFR, mL/min/1.73 m^2^	63.7 (17.6)	28.3–115.9	64.8 (16.9)	28.3–110.3	62.7 (18.3)	30.6–115.9	0.621
Albumin, g/dL	4.3 (0.4)	3.5–5.2	4.2 (0.3)	3.5–5.2	4.4 (0.3)	3.8–5.2	0.014
UACR, mg/gCr	156.7 (339.4)	1.5–1808.3	167.1 (349.2)	1.5–1705.4	147.8 (335.3)	2.7–1808.3	0.516
**Daily blood glucose level**							
05:00 h, mg/dL	116.6 (22.2)	57–254	113.5 (19.6)	57–205	119.4 (24.2)	58–254	0.485
Before breakfast, mg/dL	123.0 (21.7)	51–215	119.5 (20.4)	63–201	126.0 (22.6)	51–215	0.216
2 h after breakfast, mg/dL	180.7 (34.3)	68–383	184.7 (38.8)	68–349	177.3 (29.9)	72–383	0.880
Before lunch, mg/dL	126.7 (33.6)	43–313	137.3 (38.5)	43–313	117.6 (25.9)	45–243	0.011
Before dinner, mg/dL	134.9 (27.5)	55–331	139.2 (33.0)	60–331	131.2 (21.3)	55–267	0.339
Fluctuation, mg/dL	91.4 (28.5)	32–155	97.0 (29.7)	49–155	86.5 (26.8)	32–137	0.127
Frequency of hypoglycemia^*^	0.71 (1.3)	0–7	0.72 (1.5)	0–7	0.70 (1.2)	0–4	0.719
***Mobility function***							
Sarcopenia, *n* (%)	8 (11.6)		7 (21.9)		1 (2.7)		0.021
Low muscle mass, *n* (%)	10 (14.5)		9 (28.1)		1 (2.7)		0.004
Low grip strength, *n* (%)	24 (34.8)		17 (53.1)		7 (18.9)		0.003
Slow walking speed, *n* (%)	4 (5.8)		4 (12.5)		0 (0.0)		0.042

Data are presented as the mean (standard deviation) or as numbers and percentages. * Indicates the per-patient averages at the measuring points during the study period. **^†^** The quantitative variables age, body mass index, eGFR, daily blood glucose levels before breakfast and fluctuation were examined by unpaired t-tests, and other quantitative variables were analyzed by the Mann–Whitney U test. The categorical variables thiazolidine, insulin secretion promoter, and GLP-1 receptor agonists were examined by Fisher’s exact test, and other categorical variables were analyzed by the chi-squared test. Abbreviations: DPP4, dipeptidyl peptidase 4; eGFR, estimated glomerular filtration rate; GLP-1, glucagon-like peptide-1; HbA1c, glycated hemoglobin A1c; HDL cholesterol, high-density lipoprotein cholesterol; LDL cholesterol, low-density lipoprotein cholesterol; UACR, urine albumin-to-creatinine ratio.

**Table 2:** Association of glucose fluctuations with sarcopenia.

	Glucose Fluctuations			
	Differences *	OR	95% CI	*p*-Value
No sarcopenia	Reference			
Sarcopenia	29.3 mg/dL	1.045	(1.007; 1.083)	0.018
Normal muscle mass	Reference			
Low muscle mass	22.7 mg/dL	1.031	(1.000; 1.064)	0.0499
Normal grip strength	Reference			
Low grip strength	20.0 mg/dL	1.029	(1.006; 1.053)	0.014
Normal walking speed	Reference			
Slow walking speed	48.0 mg/dL	1.092	(1.018; 1.172)	0.014

Logistic regression with a step-wise method. The dependent variables were sarcopenia and its components. The independent variable was glucose fluctuations. All analyses were adjusted for age, HbA1c level and the presence of diabetic neuropathy. *Indicates the differences relative to glucose levels in individuals in the no sarcopenia, normal muscle mass, normal grip strength, and normal walking speed groups. Abbreviations: CI, confidence interval; OR, odds ratio.
